# Surgical Treatment of Epilepsy

**Published:** 1851-01

**Authors:** 


*Surgical Treatment of Epilepsy.*—In the *Western Lancet*, for November, Dr. Holston, of New Concord, Ohio, presents a number of interesting cases of Epilepsy, treated surgically, and we think at least two of the cases entitled to a very general reading on the part of medical practitioners. The treatment of certain forms of epilepsy, by operations upon the head has long been familiar, and a physician who may be called upon to treat epilepsy would be culpable if he left the possible pressure upon the brain, out of his investigations of the cause of the particular attack. But Dr. Holston's path does not lie altogether in this beaten range, as the reader will see in the following details:

A young lady, aged 15, came under Dr. Holston's care. Her general health was good. Several years anterior to the call on Dr. H. "she had run a piece of glass into the inner side of the plantar aspect of the left foot, and since then has been afflicted with paroxysms of epilepsy, recurring daily, and preceded by an aura, arising from the scar of the injury." The patient complained of stinging pains in the region of the cicatrix, and Dr. H. thinking some spiculæ of glass might have remained, made an incision in search of them. The wound thus made, suppurated two or three weeks, and during that time, the epilepsy was absent. As soon as the wound healed, the fits returned, which ceased upon a renewal of the wound. The complaints of the patient induced the mother to heal up the wound, aud in two months the fits returned with all their former violence. The girl is yet alive, a hopeless idiot.

CASE II. A girl aged 14, who had never menstruated, was the subject of occasional attacks of epilepsy, for two or three years, and they had lately increased in number and violence. There was an extensive necrosis of the femur, which discharged from several orifices on the inner, posterior, and outer aspect of the limb. The necrosed fragments were removed, by an operation, the case remarkably well treated, and in four weeks the wound healed; menstruation returned, the necrosis and epilepsy left together, and the patient remains well now, after a lapse of twelve years.

The next four cases belong to that familiar surgery of the head, of which we spake in the preliminary remarks. Dr. H. deserves credit for the excellent manner in which he managed these four cases, but they have not the novelty and interest that attach to the two cases, of which we have made a synopsis.

In the 7th and last case, Dr. Holston undertook the cure of a case of epilepsy, by dividing the median and ulnar nerves. This was a failure, of course, and Dr. Holston promises not to repeat such surgery.

We must beg leave to hint to our confrère of the Western Lancet, that his proof reader was sadly at fault in reading Dr. Holston's communication. B.

